# Increased Mitochondrial Protein Levels and Bioenergetics in the *Musculus Rectus Femoris* of Wfs1-Deficient Mice

**DOI:** 10.1155/2018/3175313

**Published:** 2018-11-21

**Authors:** Margus Eimre, Kalju Paju, Nadežda Peet, Lumme Kadaja, Marian Tarrend, Sergo Kasvandik, Joosep Seppet, Marilin Ivask, Ehte Orlova, Sulev Kõks

**Affiliations:** ^1^Chair of Pathological Physiology, Institute of Bio- and Translational Medicine, University of Tartu, Ravila 19, 50411 Tartu, Estonia; ^2^Proteomics Core Facility, Institute of Technology, University of Tartu, Nooruse 1, 50411 Tartu, Estonia; ^3^Department of Pathology, Tartu University Hospital, L. Puusepa 8, 51014 Tartu, Estonia

## Abstract

Wfs1 deficiency leads to a progressive loss of plasma insulin concentration, which should reduce the consumption of glucose in insulin-dependent tissues, causing a variety of changes in intracellular energy metabolism. Our objective here was to assess the changes in the amount and function of mitochondrial proteins in different muscles of Wfs1-deficient mice. Mitochondrial functions were assayed by high-resolution oxygraphy of permeabilized muscle fibers; the protein amount was evaluated by liquid chromatography tandem mass spectrometry (LC/MS/MS) analysis and mRNA levels of the uncoupler proteins UCP2 and UCP3 by real-time PCR; and citrate synthase (CS) activity was determined spectrophotometrically in muscle homogenates. Compared to controls, there were no changes in proton leak and citrate synthase activity in the heart and *m. soleus* tissues of Wfs1-deficient mice, but significantly higher levels of both of these factors were observed in the *m. rectus femoris*; mitochondrial proteins and mRNA of UCP2 were also higher in the *m. rectus femoris*. ADP-stimulated state 3 respiration was lower in the *m. soleus*, remained unchanged in the heart, and was higher in the *m. rectus femoris*. The mitochondrial protein amount and activity are higher in Wfs1-deficient mice, as are mitochondrial proton leak and oxygen consumption in *m. rectus femoris*. These changes in muscle metabolism may be important for identifying the mechanisms responsible for Wolfram syndrome and diabetes.

## 1. Introduction

Wolframin, which is encoded by the *WFS1* gene, is a transmembrane glycoprotein found primarily in the endoplasmic reticulum (ER) and is ubiquitously expressed at high levels in the brain, pancreas, and heart but also has been observed in the liver, spleen, skeletal muscles, and kidney [1, 2]. Deficiency of wolframin induces ER stress and calcium depletion in cells [3, 4], which eventually may lead to the development of Wolfram syndrome type 1 (WS), a complex disease associated with diabetes insipidus, diabetes mellitus, optic atrophy, and deafness [5]. Short stature is also quite common in patients with WS [6–8], and people with WS often suffer from chronic and progressively increasing fatigue [9], as well as muscle spasms, which are common in most patients and can be very serious [10, 11].

The development of diabetes has been associated with increased ER stress and apoptosis in pancreatic beta cells, leading to insufficient insulin secretion [3, 12]. Wolfram syndrome-related diabetes (WSD) usually develops in childhood [13, 14] and tends to occur earlier than type 1 diabetes (5.4 vs. 7.9 years of age, respectively, *P* < 0.001) [15]. Normally, approximately three-quarters of the total body glucose uptake stimulated by insulin is mediated by the skeletal muscles [16–18], and therefore, deficit of insulin or resistance of muscle fibers to insulin in diabetes mellitus causes changes in muscle energy metabolism. Diabetic patients may experience muscular weakness in the ankles and knees [19]. Hand-grip strength values were also found to be lower in diabetes patients than in age-matched control subjects [20, 21]. Muscle weakness is related to signs and severity of polyneuropathy (PN), a frequent complication for people with diabetes. In addition to PN, diabetes often leads to lower strength per unit of striated muscle and to the slowing of movements [22]. Accumulation of glycation end products may also be a cause of impaired muscle function in these patients [23].

Previous research has suggested a possible link between WS and mitochondrial DNA mutations [24], and patients with Wolfram syndrome have also been described as having heteroplasmic deletions of mtDNA in skeletal muscles [25, 26]. However, mitochondrial functioning has been poorly studied, with conflicting results [25, 27, 28]. Studies of muscle metabolism and mitochondrial function are limited by the small number of WS patients and a degree of randomness in the collection of biopsies from heterogeneous human muscles; in addition, it is also extremely difficult to analyze changes in different types of human muscle.

Several Wfs1-deficient mice models have been bred in order to study WS [3, 12, 29]. The mouse model we created exhibits a more severe phenotype than mutant mice developed by other labs and as such is a more precise model for the study of WS [30]. As Wfs1 deficiency leads to a progressive loss of *β*-cells in the pancreas and lower concentrations of plasma insulin [29, 30], our mouse model can also be used to study the mechanisms responsible for type 1 diabetes. Our objective here was to assess changes in mitochondrial function and in the amounts and enzymatic activities of mitochondrial and glycolytic pathway proteins in muscles of Wfs1-deficient mice.

## 2. Materials and Methods

### 2.1. Animals

Wfs1-deficient (Wfs1KO) mice were generated as described previously [29, 31]. Experiments were performed with 9–12-month-old Wfs1KO male mice with a 129S6/SvEvTac background and their wild-type littermates. The animals were housed under standard laboratory conditions and kept on a 12 h light–dark cycle (lights on at 07:00) and were given free access to food and water. All experiments in this study involving animals were performed in accordance with the European Parliament Directive 2010/63/EU and under permit (no. 86, May 4, 2016) from the Estonian National Board of Animal Experiments.

### 2.2. Chemicals and Reagents

K-Lactobionate, ethylene glycol-bis(*β*-aminoethyl ether)-N,N,N′,N′-tetraacetic acid (EGTA), and taurine were obtained from Fluka (Buchs, Switzerland); reduced nicotinamide adenine dinucleotide (NADH), pyruvate, and saponin were obtained from SERVA (Heidelberg, Germany); MgCl_2_ and KCl were obtained from ACROS (Geel, Belgium); and leupeptin was obtained from Roche (Basel, Switzerland). All other reagents, ADP, ATP, bovine serum albumin, enzymes (glucose-6-phosphate dehydrogenase and lactate dehydrogenase), dithiothreitol, KH_2_PO_4_, 4-(2-hydroxyethyl)-1-piperazineethanesulfonic acid (HEPES), L-glutamate, DL-malate, rotenone (Rot), succinate, atractyloside, antimycin A, N,N,N′,N′-tetramethyl-p-phenylenediamine (TMPD), ascorbate, ethylenediaminetetraacetic acid (EDTA), glucose, Triton X-100, Tris, phosphoenolpyruvate (PEP), and NADP were obtained from Sigma (St. Louis, MO, United States).

### 2.3. Determination of Activities of the Respiratory Chain Complexes

Mice received 5 units/g b.wt. of heparin intraperitoneally and were killed by cervical dislocation. The heart, *m. soleus*, and white glycolytic section of the *m. rectus femoris* were excised and placed into ice-cold saline to ensure rapid cooling and blood removal. Thin muscle bundles from muscles were then excised in ice-cold solution A, which contained 2.77 mM CaK_2_EGTA, 7.23 mM K_2_EGTA 7.23 (free calcium concentration 0.1 *μ*M), 6.56 mM MgCl_2_, 0.5 mM dithiothreitol (DTT), 50 mM potassium 2-(N-morpholino)-ethanesulfonate (K-MES), 20 mM imidazole, 20 mM taurine, 5.3 mM ATP, and 15 mM phosphocreatine, at pH 7.1 adjusted at 4°C. Muscle fibers were separated using sharp-ended needles, leaving only small areas of contact; the fibers were then transferred to vessels filled with solution A, to which an additional 50 *μ*g of saponin per ml was added, and incubated with mild stirring for 30 min at 4°C for complete solubilization of the sarcolemma. Skinned (permeabilized) fibers were washed three times in solution B, which contained 0.5 mM EGTA, 3 mM MgCl_2_, 60 mM K-lactobionate, 20 mM taurine, 3 mM KH_2_PO_4_, 20 mM HEPES, 110 mM sucrose, and 5 mM pyruvate, and 1 mg/ml bovine serum albumin (BSA) and was set at pH 7.1 (adjusted at 25°C) for 10 min at 4°C by stirring to completely remove all metabolites, particularly trace amounts of ADP.

The activities of the mitochondrial respiratory chain segments in permeabilized muscle fibers were assessed using the polarographic method (Oroboros, Innsbruck, Austria) as respiration rates (VO_2_) in solution B with 10 mM pyruvate or glutamate, 2 mM malate, and 0.2 *μ*M free Ca^2+^ at 25°C. Following registration of mitochondrial respiration under nonphosphorylating conditions (basal respiration rate [*V*_0_]) in the oxygraph chamber, 2 mM ADP was added to monitor the maximum rate of NADH-linked ADP-dependent respiration; subsequently, complex I was inhibited by 10 *μ*M of rotenone (Rot). Then, 10 mM succinate was added to activate respiratory chain complex II-dependent respiration, 0.1 mM atractyloside (Atr) was added to assess respiratory control by adenine nucleotide translocase (ANT), 10 *μ*M antimycin A was added to inhibit complex III and thereby block the electron flow from complex II to cytochrome c, and 0.5 mM tetramethylphenylene diamine (TMPD) with 2 mM ascorbate was added to activate cytochrome oxidase (COX). Activation of COX in the case of blocked oxidative phosphorylation (OXPHOS) in the presence of Atr was possible due to the smaller number of protons pumped by the respiratory chain per oxygen consumed [32]. In this case, the proton leak in the presence of TMPD was no longer a limiting factor for the respiratory chain. Finally, 5 mM sodium azide (NaN_3_) was added to inhibit COX. Antimycin-sensitive respiration in the presence of atractyloside (*V*_Atr_–*V*_Ant_) was considered to represent the proton leak. The NaN_3_-sensitive portion of respiration with TMPD and ascorbate (*V*_TMPD_–*V*_NaN3_) exhibits COX-related respiration.

### 2.4. Gene Expression Study

The *rectus femoris* muscles used in the gene expression analysis were suspended in RNAlater RNA Stabilization Reagent (Qiagen, Düsseldorf, Germany). Total RNA was isolated using the RNeasy Mini Kit (Qiagen). Genomic DNA wipeout and reverse transcription were performed with QuantiTect® Reverse Transcription Kit (Qiagen), in accordance with the manufacturer's instructions (QuantiTect® Reverse Transcription Handbook, 2005). A QuantiTect SYBR Green PCR Kit (Qiagen, Düsseldorf, Germany) was used to perform real-time PCR amplification with mouse gene-specific primers (Table 1). Fluorescence data during PCR was collected with a StepOnePlus™ Real-Time PCR Instrument (Applied Biosystems, Foster City, CA, United States) and an intercalator-based approach (also known as the SYBR Green method). The comparative threshold cycle (ΔC_T_, ΔΔC_T_) technique was used to determine the relative target quantities in samples [33], with all measurements normalized to the endogenous control gene ACTB. Relative target quantity in each sample was assessed by comparing normalized target quantity in each sample to normalized target quantity in the reference sample.

### 2.5. Determination of Enzymatic Activities

The heart muscle, *m. soleus*, and the glycolytic part of *m. rectus femoris* were frozen in liquid nitrogen and stored at −80°C; these muscle tissues were later allowed to thaw at 0°C and homogenized by sonication (Bandelin Sonopuls HD 2200, probe MS 72, Sigma-Aldrich, St. Louis, MO, United States) on ice for 15 s in a medium containing 1 mM EDTA, 1 mM DTT, 10 mM glucose, 5 mM MgCl2, and 5 mM HEPES, at pH 8 (maintained by NaOH), with 0.1% Triton X-100 and 5 mg/ml leupeptin added to the mixture. After homogenization, the probe was kept on ice for an additional 30 s. The homogenization cycle was repeated twice thereafter, and the homogenates were left on ice for 1 h to allow complete extraction of the enzymes. Measurement of pyruvate kinase (PK) activity was performed using a spectrophotometric cuvette under stirring conditions in solution containing 26.7 mM KHPO_4_ 26.7 (pH 7.6, 37°C), 6.67 mM MgSO_4_, 0.24 mM NADH, 0.5 mM PEP, 3 mM ADP, and 10 IU/ml lactate dehydrogenase (LDH). After baseline registration, the reaction was initiated with the addition of the homogenate, and PK activity was derived from subsequent changes in NADH oxidation rates at 340 nm. Measurement of citrate synthase (CS) activity was performed using a Citrate Synthase Assay Kit (Sigma-Aldrich, St. Louis, MO, United States).

### 2.6. Proteomics Sample Preparation

Protein content of the homogenates was determined by precipitation with a methanol : chloroform : water solution in a 2 : 1 : 3 ratio, respectively, and suspended in digestion buffer (7 M thiourea, 2 M urea, and 100 mM ammonium bicarbonate). After reduction with 2.5 mM dithiothreitol and alkylation with 5 mM chloroacetamide, the samples were first predigested with 1 : 50 (protease : protein ratio) Lys-C (Wako Pure Chemical Industries, Osaka, Japan) for 4 h, diluted 5-fold with 100 mM ammonium bicarbonate, and then digested for 16 h with 1 : 50 trypsin at room temperature. Peptides were desalted using C18 (3M, Maplewood, Minnesota, United States) StageTips and reconstituted in 0.5% trifluoroacetic acid.

### 2.7. LC/MS/MS Analysis

Injected peptides were separated on an Ultimate 3000 RSLCnano system (Dionex, Sunnyvale, California, United States) using a C18 cartridge trap column (Dionex) in backflush configuration and an in-house packed (3 *μ*m C18 particles, Dr. Maisch GmbH, Ammerbuch, Germany) analytical 50 cm × 75 *μ*m emitter column (New Objective Inc., Woburn, MA, United States). Peptides were eluted at 200 nl/min with a 5–35% B 180 min gradient (buffer B: 80% acetonitrile + 0.1% formic acid, buffer A: 0.1% formic acid) to a Q Exactive Plus (Thermo Fisher Scientific, Waltham, MA, United States) tandem mass spectrometer operating with a top 10 strategy. Briefly, one 350–1400 m/z MS scan at a resolution of *R* = 70,000 was followed by higher energy collisional dissociation fragmentation (normalized collision energy of 26) of the 10 most intense ions (charge states +2 to +6) at *R* = 17,500. MS and MS/MS ion target values were 3*e*6 and 5*e*4, respectively, and injection times were limited to 50 ms. Dynamic exclusion was set to 50 s.

### 2.8. Proteomics Data Analysis

Mass spectrometric raw data were processed with MaxQuant 1.5.3.17 software [34]. Label-free quantification with the MaxQuant LFQ algorithm [35] was enabled with default settings with the exception of the minimal ratio count (number of peptides quantified to report a protein quantification), which was set to 1 peptide. Methionine oxidation and protein N-terminal acetylation were set as variable modifications. Cysteine carbamidomethylation was defined as a fixed modification. Search was performed against the UniProt (http://www.uniprot.org) *Mus musculus* reference proteome database (downloaded on 11 November 2015; 57,320 entries) using the tryptic digestion rule Trypsin/P. Only identifications minimally 1 peptide 7 amino acids long were accepted, and transfer of identifications between runs was enabled. Peptide-spectrum match and protein false discovery rate (FDR) were kept below 1% using a target-decoy approach. All other parameters were kept at default settings.

### 2.9. Statistical Analysis

All statistical analyses were performed with Student's *t*-test, and data are presented as mean ± SEM, with significance set at *P* < 0.05.

## 3. Results

### 3.1. Anatomical Data of Wfs1-Deficient and Wild-Type Mice

Body weights and muscle weights of Wfs1-deficient mice were significantly reduced compared to wild-type mice (Table 2); however, the heart-to-body weight and *soleus* muscle-to-body weight ratios were similar in both mice, suggesting that the absence of wolframin was not associated with atrophy of these muscles. In contrast, the *quadriceps femoris* muscle-to-body weight ratio was significantly reduced, indicating atrophy of this muscle and in part of the *m. rectus femoris*.

### 3.2. Mitochondrial Respiration in Muscles of Wfs1-Deficient Mice

**Figure 1:**
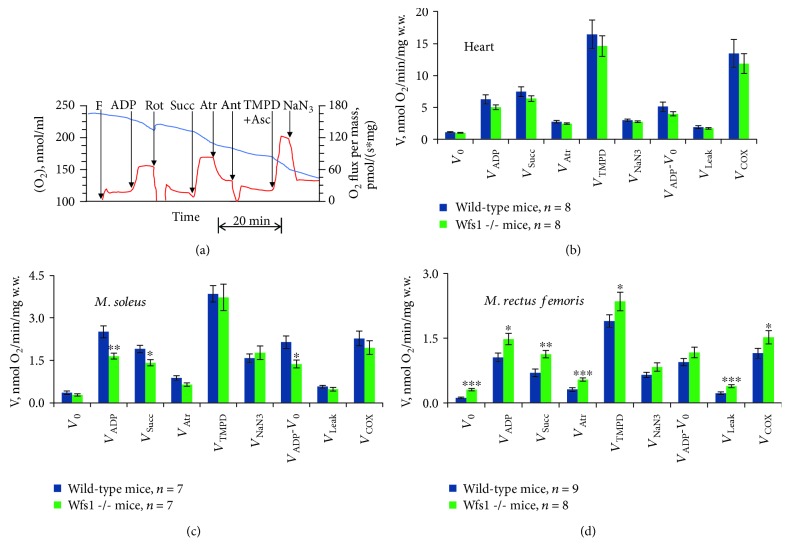
(a) Original recording of the assessment of the respiratory chain function in skinned muscle fibers. Additions: F: fibers; ADP: 2 mM ADP; Rot: 10 *μ*M rotenone; Succ: 10 mM succinate; Atr: 0.1 mM atractyloside; Ant: 10 *μ*M antimycin A; TMPD + Asc: 0.5 mM TMPD with 2 mM ascorbate; NaN_3_: 5 mM sodium azide. Upper, blue line: [O_2_]; lower, red line: O_2_ flux. Summary of results, which correspond to the experimental scheme shown in (a), is given in groups of permeabilized muscle fibers from the heart (b), *musculus soleus* (c), and *musculus rectus femoris* (d). *V*_0_: basal respiration; *V*_ADP_: complex I- and ADP-dependent respirations; *V*_Succ_: complex II-dependent respiration in the presence of rotenone with succinate; *V*_Atr_: atractyloside-insensitive respiration rate; *V*_TMPD_: respiration with TMPD and ascorbate in the presence of antimycin A; *V*_NaN3_: respiration in the presence of sodium azide; *V*_Leak_: the difference between *V*_Atr_ and *V*_Ant_, proton leak-dependent respiration; *V*_COX_: sodium azide-sensitive portion of the respiration rate in the presence of TMPD and ascorbate. Data are mean ± SEM. Compared to wild type: ^∗^ *P* < 0.05, ^∗∗^ *P* < 0.01, and ^∗∗∗^ *P* < 0.001.

The function of different respiratory chain complexes in the muscle fibers of saponin-skinned wild-type and Wfs1-deficient mice was assessed using a substrate/inhibitor titration protocol [36, 37] (Figure 1(a)). Mitochondrial respiration in the permeabilized muscle fibers increased over the basal levels following the addition of ADP, indicating a coupling of complex I-dependent respiration to ADP phosphorylation. After registration of respiration in the presence of ADP (Figure 1(a)), rotenone was added to inhibit complex I, and succinate was subsequently added to stimulate complex II-dependent respiration. Next, atractyloside was added, which strongly reduces succinate-dependent respiration; this was suppressed even further by the addition of 10 *μ*M antimycin A, which inhibits the electron flow from complex III to cytochrome c in mitochondria. Cytochrome c oxidase was activated with TMPD and ascorbate and then suppressed by NaN_3_ (Figure 1(a)). As previous experiments have shown, *V*_NaN3_ remains higher than *V*_0_ [38, 39], a background respiration caused by strong autooxidation of TMPD [40]. We found that the basal respiration rate (*V*_0_) was 2.59 times (*P* < 0.001) greater in Wfs1-deficient *m. rectus femoris* (Figure 1(d), Table S1) than in wild-type *m. rectus femoris*, whereas there were no differences in the heart and *m. soleus* between the two mice (Figures 1(b) and 1(c)). After the addition of ADP into the medium of wild-type *m. rectus femoris*, the rate of respiration increased to 1.06 ± 0.10 nmol O_2_/min/mg w.w. (Figure 1(d), Table S1), a rate lower than that of the corresponding increases in the other muscles. However, for the respiratory control index, the relative increase of 10.6 times with respect to *V*_0_ for the wild-type *m. rectus femoris* was greater than that for the other muscles (Table S1). The corresponding relative increase of the respiration rate for the Wfs1-deficient *m. rectus femoris* was only 4.87 times, which was lower than in the *m. soleus* and practically the same as in the heart. *Musculus rectus femoris V*_ADP_ was 40% higher in Wfs1-deficient mice than in wild-type mice (*P* < 0.01) (Figure 1(d)) but was 34% lower in Wfs1-deficient *m. soleus* than in wild-type *m. soleus* (*P* < 0.01) (Figure 1(c)). In addition, ADP- and complex II-dependent respirations were 62% higher in Wfs1-deficient *m. rectus femoris* (*P* < 0.001), whereas Wfs1-deficient *m. soleus* was 24% lower than in wild-type *m. soleus* (*P* = 0.012). Blocked OXPHOS (Atr) respiration was 75% higher in Wfs1-deficient *m. rectus femoris* than in wild-type *m. rectus femoris* (*P* < 0.001). The difference between *V*_Atr_ and *V*_Ant_, as proton leak-dependent respiration, was 73% greater in Wfs1-deficient *m. rectus femoris* than in wild-type *m. rectus femoris* (*P* = 0.001), and complex IV-dependent respiration (*V*_COX_) was greater by 31% in Wfs1-deficient *m. rectus femoris*. There were no significant differences in complex I and OXPHOS-related oxygen flux (*V*_ADP_-*V*_0_) between wild-type and Wfs1-deficient *m. rectus femoris* (0.936 ± 0.08 and 1.171 ± 0.10 nmol O_2_/min/mg w.w., respectively).

### 3.3. Quantitative Real-Time RT-PCR Data Analysis

**Figure 2:**
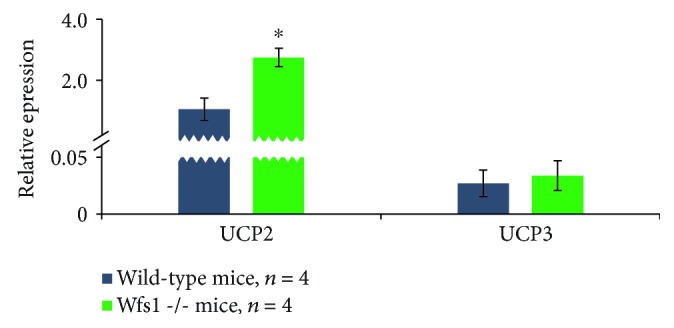
Relative expression of uncoupler proteins in homogenates of muscles in relation to housekeeping gene ACTB. ^∗^ *P* < 0.05.

It is possible that the greater basal respiration (*V*_0_) and differences in *V*_Atr_ and *V*_Ant_ in Wfs1-deficient *m. rectus femoris* compared to wild-type *m. rectus femoris* that we observed in the respiratory experiments were caused by increased proton leak in this muscle. Proton leak in skeletal muscles depends on uncoupler protein levels, and thus, we examined UCP2 and UCP3 expressions at the genetic level. We found that, relative to the housekeeping gene ACTB, UCP2 mRNA levels were 1.05 ± 0.37 in wild-type *m. rectus femoris* and 2.75 ± 0.30 in Wfs1-deficient *m. rectus femoris*, a difference of 2.6 times (*P* < 0.05). UCP3 expression was an order of magnitude lower than that of UCP2 and did not differ among the muscle types (Figure 2).

### 3.4. Enzymatic Activity

**Figure 3:**
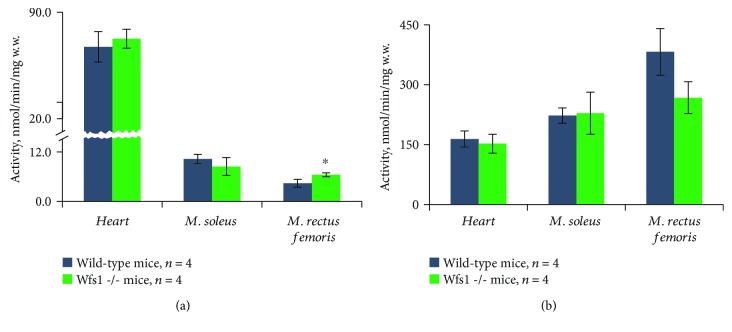
Citrate synthase (a) and pyruvate kinase (b) activity. Compared to wild type: ^∗^ *P* < 0.05.

Citrate synthase activity was 77.0 ± 10.0 in wild-type heart muscles, but activity of this enzyme was 7.5 times lower (10.3 ± 1.1) in the *m*. *soleus* and 2 times lower still (4.4 ± 0.9) in the *m. rectus femoris* than in the *m*. *soleus* (Figure 3(a)). Compared with controls, there was no change in citrate synthase activity levels in Wfs1-deficient heart and *m*. *soleus* fibers, but activity levels were 1.46-fold (*P* < 0.05) higher in the Wfs1-deficient *m. rectus femoris*. Pyruvate kinase activity levels did not differ between wild-type and Wfs1-deficient muscles (Figure 3(b)).

### 3.5. Proteomics of Mitochondrial Proteins

**Figure 4:**
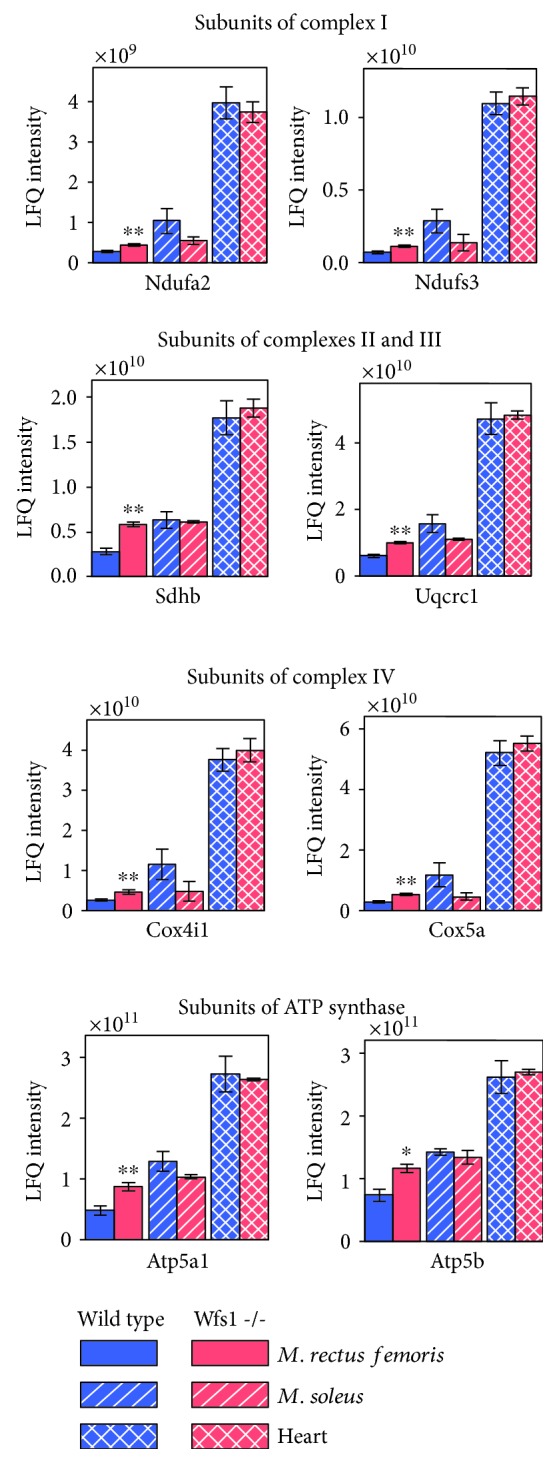
Amounts of some subunits of mitochondrial respiratory chain complexes. Ndufa2: NADH dehydrogenase [ubiquinone] 1 alpha subcomplex subunit 2; Ndufs3: NADH dehydrogenase [ubiquinone] iron-sulfur protein 3; SDHB: succinate dehydrogenase [ubiquinone] iron-sulfur subunit; Uqcrc1: cytochrome b-c1 complex subunit 1; Cox4i1: cytochrome c oxidase subunit 4 isoform 1; Cox5a: cytochrome c oxidase subunit 5A; Atp5a1: ATP synthase subunit alpha; Atp5b: ATP synthase subunit beta. Compared to wild type: ^∗^ *P* < 0.05 and ^∗∗^ *P* < 0.01.

We performed an LC/MS/MS analysis to confirm the results obtained from the oxygraphic experiments. Twenty-seven subunits of respiratory chain complex I were identified. In wild-type mice, protein amounts were determined to be ~5 times higher in the *m*. *soleus* than in the *m. rectus femoris* and ~5 times higher in the heart muscle than in the *m. soleus* (Figure S1). Amounts of typical representatives of the complex I proteins NADH dehydrogenase [ubiquinone] 1 alpha subcomplex subunit 2 (Ndufa2) and NADH dehydrogenase [ubiquinone] iron-sulfur protein 3 (Ndufs3) are shown in Figure 4. Respiratory chain complex II or succinate dehydrogenase is composed of four subunits consisting of the succinate dehydrogenase [ubiquinone] flavoprotein subunit SDHA, iron-sulfur protein SDHB, cytochrome b560 subunit SDHC, and cytochrome b small subunit SDHD. This enzyme plays a role in both the citric acid cycle and the respiratory chain. Our LC/MS/MS analysis revealed the same increases (2.08 times) in both hydrophilic subunits SDHA and SDHB in Wfs1-deficient *m. rectus femoris* compared to wild-type *m. rectus femoris* and that the amounts of two hydrophobic subunits of succinate dehydrogenase, SDHC and SDHD, were more than an order of magnitude lower and did not differ significantly between wild-type and Wfs1-deficient muscles (Figure S4). Amounts of complex II protein and succinate dehydrogenase [ubiquinone] iron-sulfur subunit in wild-type and Wfs1-deficient mice muscles are shown in Figure 4. Eight components of the ubiquinol-cytochrome c reductase complex (complex III) were identified in Wfs1-deficient *m. rectus femoris*, of which amounts of six components were significantly higher by ~1.5 times (Figure S2). Similar results were obtained for respiratory chain complex IV, for which protein amounts were significantly higher in Wfs1-deficient *m. rectus femoris* than in wild-type *m. rectus femoris* (Figure S2). Amounts of typical representatives of complex IV are shown in Figure 4. Mitochondrial membrane ATP synthase (F_1_F_0_ ATP synthase or complex V) consists of two structural domains, subunits alpha (Atp5a1) and beta (Atp5b), which form the catalytic core in the F1 domain, which were compared to other subunits of F_1_F_0_ ATP synthase, and which are most abundant in muscle homogenates (Figure S3). The amounts of these subunits are shown in Figure 4. Abundance of subunit alpha Atp5a1 was 1.81 times (*P* = 0.008) and that of subunit beta 1.58 times (*P* = 0.016) higher in Wfs1-deficient *m. rectus femoris* than in wild-type *m. rectus femoris*. In the homogenates, H^+^ transporting delta subunit of F1 domain Atp5d; ATP synthase subunit gamma Atp5c1 of the central stalk; and several subunits of ATP synthase F0 domain, namely, d Atp5h, e Atp5i, f Atp5j2, g Atp5l, o Atp5o, and ATP synthase-coupling factor 6 Atp5j, were also all detected at moderate levels. The amounts of all these subunits were higher in the *m. rectus femoris* of Wfs1-deficient mice than in that of wild-type mice (Figure S3).

**Figure 5:**
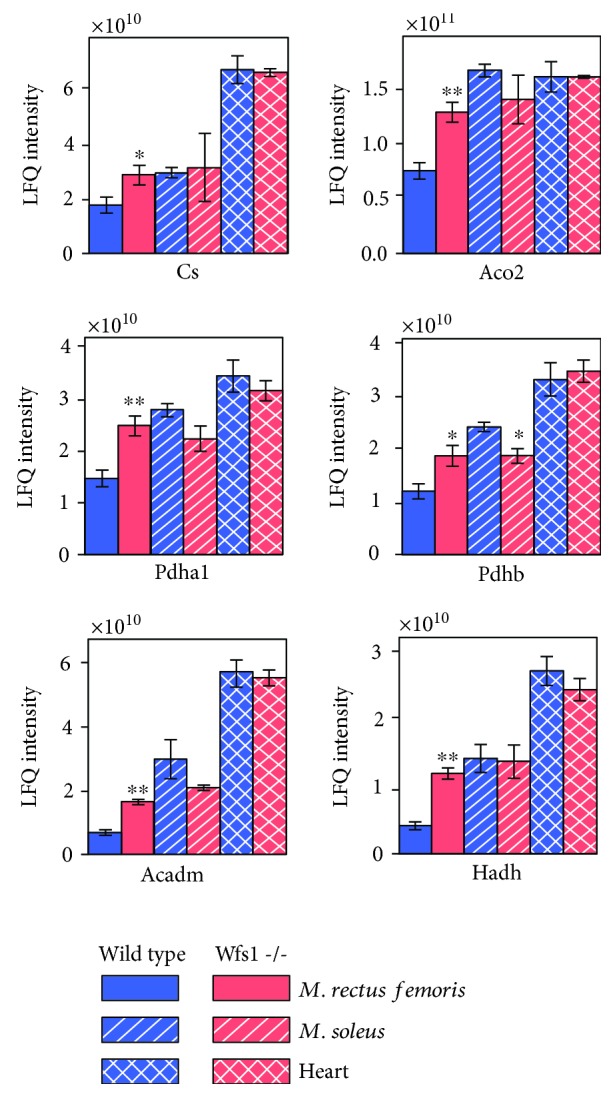
Amounts of some mitochondrial proteins and their subunits. Cs: citrate synthase; Aco2: aconitate hydratase; Pdha1: pyruvate dehydrogenase E1 component subunit alpha; Pdhb: pyruvate dehydrogenase E1 component subunit beta; Acadm: medium-chain specific acyl-CoA dehydrogenase; Hadh: hydroxyacyl-coenzyme A dehydrogenase. Compared to wild type: ^∗^ *P* < 0.05 and ^∗∗^ *P* < 0.01.

The citric acid cycle is also an important mitochondrial enzymatic system that provides electrons to the respiratory chain. Citrate synthase, the first step of the citric acid cycle, is commonly used as a quantitative enzyme marker for the mitochondrial presence. As with enzyme activity, higher amounts of citrate synthase were present in Wfs1-deficient *m. rectus femoris* compared to wild-type *m. rectus femoris* (by 1.62 times, *P* < 0.05, Figure 5), as was also found for aconitate hydratase Aco2 (by 1.71 times, *P* = 0.001), isocitrate dehydrogenase [NAD] subunit gamma Idh3g (by 1.86 times, *P* = 0.008), 2-oxoglutarate dehydrogenase Ogdh (by 1.42 times, *P* < 0.05), succinyl-CoA ligase subunit beta (Sucla2) (by 1.42 times, *P* < 0.05), and mitochondrial malate dehydrogenase (by 1.93 times, *P* = 0.001) (Figure S4). Concentrations of pyruvate dehydrogenase, an enzyme closely linked to the citrate cycle, were also increased in Wfs1-deficient *m. rectus femoris* compared to wild-type *m. rectus femoris*, with pyruvate dehydrogenase E1 component subunit alpha 1.69 times (*P* = 0.0021) and subunit beta 1.56 times (*P* = 0.018) higher. Beta-oxidation of fatty acids takes place in the mitochondria, and all enzymes involved in beta-oxidation that were identified in our LC/MS/MS analysis were also significantly higher (1.7–3.3 times, *P* < 0.05) in Wfs1-deficient *m. rectus femoris* than in wild-type *m. rectus femoris* (Figure S5). Amounts of the typical representative medium-chain-specific acyl-CoA dehydrogenase (Acadm) and hydroxyacyl-coenzyme A dehydrogenase (Hadh) are also shown in Figure 5.

### 3.6. Amounts of Indicators of Metabolic Profile and Regulators of Mitochondrial Function

Sirtuins are a class of proteins of relatively small molecular mass; these proteins have deacylase activity and regulate important biological pathways [41]. Mitochondrial sirtuin 3 and sirtuin 5 amounts were 6.24 and 1.68 times higher, respectively, in Wfs1-deficient *m. rectus femoris* than in wild-type *m. rectus femoris* (Table 3), and sirtuin 5 was considerably more abundant than sirtuin 3 in all muscles. Amounts of cytoplasmic sirtuin 2 were lower than those of mitochondrial sirtuins in oxidative muscles. Both parvalbumin, a calcium-binding albumin protein, and sarcoplasmic reticulum calcium ATPase are normally found in greater quantities in fast-contracting glycolytic muscles. Parvalbumin alpha and sarcoplasmic reticulum calcium ATPase were 2.15 (*P* = 0.01) and 1.89 (*P* = 0.0006) times lower, respectively, in Wfs1-deficient *m. rectus femoris* than in wild-type *m. rectus femoris* (Table 3). Moreover, compared to the wild type, the abundance of some glycolytic enzymes was also lower in Wfs1-deficient *m. rectus femoris*; for instance, fructose-bisphosphate aldolase (Aldoa), alpha-enolase (Eno1), and L-lactate dehydrogenase A chain (Ldha) were reduced by 25% (*P* = 0.003), by 27% (*P* = 0.0163), and by 33% (*P* = 0.0042), respectively, in the *m. rectus femoris* of Wfs1-deficient mice relative to that of wild-type mice (Figure S6).

## 4. Discussion

To the best of our knowledge, this represents the first study in which the mitochondrial function and abundance of mitochondrial proteins in the muscles of Wfs1-deficient mice were characterized. Surprisingly, we found that, compared to the wild type, the amounts of the majority of mitochondrial proteins and activity levels of citrate synthase were significantly higher in Wfs1-deficient glycolytic muscle tissues (~1.5-fold). ADP-dependent respiration with complex I and II substrates was also higher in Wfs1-deficient *m. rectus femoris* than in wild-type *m. rectus femoris*, and complex IV-dependent respiration was one-third greater in this muscle in Wfs1-deficient mice than in wild-type mice (Figure 1(d)). As can be seen in Table 2, the quadriceps muscle-to-body weight ratio was lower in Wfs1-deficient mice. Similarly, STZ-induced type I diabetes produces profound atrophy of fast-twitch muscles and especially the fast glycolytic (FG) fibers [42–46]. Atrophy of glycolytic muscle fibers in our model mice most likely caused even higher rates of respiration and mitochondrial protein abundance in the Wfs1-deficient glycolytic *rectus femoris* muscle. Comparable results were reported from recent work, in which it was shown that basal oxygen consumption (measured at the whole-organism level) was 1.3 times higher in Wfs1-deficient mice, and heat production was also 1.3 times higher in Wfs1-deficient mice [47]. Transient receptor potential melastatin 8 (TRPM8) channels, which are activated by cold (8–28°C) and by some chemicals like menthol, are key pathways for heat production signaling [48–50]. Dietary menthol was shown to significantly increase oxygen consumption in WT mice but not in TRMP8KO mice, suggesting that dietary menthol increases the resting metabolic rate via TRPM8 activation [51]. Likewise, RNA sequencing analysis revealed increased expression of TRMP8 in the hippocampus of Wfs1-deficient mice, and metabolic studies have shown higher response to menthol administration in Wsf1-deficient mice compared to WT mice. Overexpressed TRMP8 in Wfs1-deficient mice might be the reason for the higher basal oxygen consumption and heat production in this type compared to WT mice [47]. Given that TRMP8-dependent thermogenesis is caused by activation of uncoupler protein 1 (UCP1) in brown adipose tissue [51], the enhanced heat production and oxygen consumption of Wfs1-deficient mice may be due to increased mitochondrial proton leak in BAT, but the results of our study suggest that elevated mitochondrial proton leak also occurs in the glycolytic muscle *rectus femoris*. Compared to the wild type, basal respiration without ADP was 2.5-folds higher and proton leak-dependent respiration 1.7-folds higher in Wfs1-deficient muscles. OXPHOS-related oxygen flux (*V*_ADP_-*V*_0_) did not differ significantly between wild-type and Wfs1-deficient *m. rectus femoris* muscles; thus, in the latter, the proportion of proton leak to OXPHOS was increased, and accordingly, the respiratory control index was 2 times lower in Wfs1-deficient *m. rectus femoris*. Increases in the relative proportion of proton leak in glycolytic skeletal muscle may depend on higher levels of UCP2, as we observed an increase in RNA levels of this protein; however, LC/MS/MS analysis failed to detect the presence of UCP2 proteins in any of the muscle types, indicating that increased proton leak simply damages mitochondria in Wfs1-deficient glycolytic muscles and that the increase in mitochondrial proteins is most likely compensating for decreasing mitochondrial quality. It has been shown that SIRT3 stimulates mitochondrial biogenesis [52–54], and thus, the higher levels of mitochondrial sirtuin 3 in Wfs1-deficient *m. rectus femoris* compared to wild-type *m. rectus femoris* likely stimulate mitochondrial biogenesis in this tissue. Concentrations of sirtuin 5 were also higher in Wfs1-deficient *m. rectus femoris*; like other sirtuins, sirtuin 5 has deacylase activity and is capable of removing acetyl, succinyl, and malonyl groups from the lysine residues of proteins, but in contrast to sirtuin 3, it does not seem to be involved in mitochondrial biogenesis [55, 56]. Levels of cytoplasmic sirtuin 2 were roughly the same in Wfs1-deficient and wild-type *m. rectus femoris*, which may simply reflect a reduction in the amount of cytoplasm relative to mitochondria as levels of mitochondrial sirtuins and other mitochondrial proteins increased.

In contrast to glycolytic *rectus femoris*, either there were no changes in the oxidative muscles of Wfs1-deficient mice or changes occurred in the opposite direction. In the heart muscle, for instance, mitochondrial protein abundance, citrate synthase activity, and mitochondrial respiration remained unchanged, and compared to the wild type, ADP-dependent respiration involving both complex I and complex II substrates was lower in Wfs1-deficient *m. soleus*. Furthermore, although we observed downward trends in the parameters of several mitochondrial proteins in Wfs1-deficient *m*. *soleus*, the amounts of other proteins and the activity level of citrate synthase did not change.

The Wfs1 protein interacts with sarco/endoplasmic reticulum ATPase (Atp2a) and negatively regulates Atp2a turnover, possibly via proteasome-mediated degradation. In Wfs1-depleted cells, Wfs1–Atp2a interaction is limited, and therefore, the amount of Atp2a is upregulated [57]; however, we did not observe higher levels of Atp2a in Wfs1-deficient muscles. In fact, Atp2a was lower in Wfs1-deficient *m. rectus femoris* than in wild-type *m. rectus femoris*, as was parvalbumin alpha (Pvalb), a protein involved in calcium signaling. Concentrations of parvalbumin are normally high in fast-contracting muscles and low in slow-contracting muscles [58], whereas slow-twitch muscles generally contain more mitochondria and less cytoplasm. We also found that, compared to the wild type, concentrations of some glycolytic enzymes were lower in Wfs1-deficient *m. rectus femoris*. As such, the reduced amounts of Atp2a1 and Pvalb may indicate a shift from fast-contracting glycolytic *m. rectus femoris* to slower oxidative muscles in Wfs1-deficient mice. Like type 1 diabetes, this shift in the glycolytic muscle type is probably triggered by insulin deficiency and the atrophy of FG fibers in Wsf1-deficient mice and missed in oxidative muscles [45, 46]. Increased relative proton leak, which reduces the efficiency of ATP synthesis, is another alteration in *m. rectus femoris* metabolism. It is possible that these same pathologies occur in patients suffering from WS; considering the high percentage of glycolytic muscles in the human body, such changes may be major causes of the chronic progressive fatigue and other muscle disorders experienced by WS patients [9].

## 5. Conclusions

Mitochondrial protein concentrations, citrate synthase activity, and mitochondrial respiration of permeabilized fibers were found to be significantly elevated in Wfs1-deficient *m. rectus femoris*. Compared to the wild type, efficiency of ATP synthesis declined in the mitochondria of Wfs1-deficient *m. rectus femoris* muscle due to higher proton leak-dependent respiration, whereas there was no difference in OXPHOS-dependent respiration.

## Figures and Tables

**Table 1:** Primers for real-time PCR analysis.

Primer	Sequence 5′ > 3′
UCP2	F: GCTGGTGGTGGTCGGAGAT
	R: TGAAGTGGCAAGGGAGG
UCP3	F: GCTGACACGAGAAACTGAACTAAA
	R: GGAGTTGACTCTGGTTTTCTTTGT
ACTB	F: AGCCATGTACGTAGCCATCCA
	R: GACTTTGCTTTCCTTGGTCAGG

F: forward; R: reverse.

**Table 2:** Anatomical characteristics of Wfs1-deficient and wild-type mice.

Characteristic	WT (*n* = 5)	Wfs1KO (*n* = 5)
Body weight (g)	30.9 ± 1.1	19.9 ± 1.2^∗∗∗^
Heart weight (mg)	161.5 ± 8.3	113.4 ± 7.6^∗∗^
Heart/body weight ratio (mg/g)	5.59 ± 0.34	6.05 ± 0.06
*Soleus* muscle weight (mg)	5.47 ± 0.28	4.50 ± 0.41^∗^
*Soleus* muscle/body weight ratio (mg/g)	0.189 ± 0.006	0.235 ± 0.020
*Quadriceps femoris* muscle weight (mg)	196.7 ± 5.93	100.3 ± 3.62^∗∗∗^
*Quadriceps femoris* muscle/body weight ratio (mg/g)	6.05 ± 0.09	4.82 ± 0.27^∗∗^

Means ± SEM are shown. Compared to wild type: ^∗^ *P* < 0.05, ^∗∗^ *P* < 0.01, and ^∗∗∗^ *P* < 0.001.

**Table 3:** LFQ intensities of proteins determined by LC/MS/MS analysis.

Proteins	Genes	*M. rectus femoris*		*M. soleus*		Heart	
		Wfs1+/+ *n* = 8	Wfs1−/− *n* = 5	Wfs1+/+ *n* = 3	Wfs1−/− *n* = 2	Wfs1+/+ *n* = 4	Wfs1−/− *n* = 4
NAD-dependent protein deacylase sirtuin-5, mitochondrial	Sirt5	1.86*E* + 08 ± 2.17*E* + 07	3.13*E* + 08 ± 3.37*E* + 07^∗∗^	4.05*E* + 08 ± 3.42*E* + 07	2.61*E* + 08 ± 1.15*E* + 08	8.24*E* + 08 ± 8.25*E* + 07	7.94*E* + 08 ± 3.06*E* + 07
NAD-dependent protein deacetylase sirtuin-3	Sirt3	3.81*E* + 06 ± 1.24*E* + 06	2.38*E* + 07 ± 6.73*E* + 06^∗∗^	7.37*E* + 06 ± 4.10*E* + 06	8.22*E* + 06 ± 7.12*E* + 06	1.66*E* + 08 ± 6.67*E* + 07	2.83*E* + 08 ± 3.61*E* + 07
NAD-dependent protein deacetylase sirtuin-2	Sirt2	4.40*E* + 08 ± 3.34*E* + 07	3.39*E* + 08 ± 3.68*E* + 07	2.44*E* + 08 ± 1.94*E* + 07	2.17*E* + 08 ± 1.03*E* + 08	5.80*E* + 07 ± 5.74*E* + 06	6.03*E* + 07 ± 1.11*E* + 07
Parvalbumin alpha	Pvalb	4.30*E* + 11 ± 5.90*E* + 10	2.00*E* + 11 ± 2.24*E* + 10^∗^	7.25*E* + 10 ± 6.04*E* + 10	5.54*E* + 10 ± 4.22*E* + 10	1.87*E* + 07 ± 1.43*E* + 07	0.00
Sarcoplasmic/endoplasmic reticulum calcium ATPase	Atp2a	3.86*E* + 11 ± 2.76*E* + 10	2.04*E* + 11 ± 1.84*E* + 10^∗∗∗^	1.25*E* + 11 ± 7.08*E* + 10	3.00*E* + 10 ± 1.42*E* + 10	2.64*E* + 10 ± 4.81*E* + 09	2.85*E* + 10 ± 2.16*E* + 09

Values are means ± SEM; *n* = the number of specimens studied. Compared to wild type: ^∗^ *P* < 0.05, ^∗∗^ *P* < 0.01, and ^∗∗∗^ *P* < 0.001.

## Data Availability

The mass spectrometry proteomics data used to support the findings of this study have been deposited to the ProteomeXchange Consortium via the PRIDE [59] partner repository with the dataset identifier PXD011019 and will be published as data article [60]. The other data used to support the findings of this study are available from the corresponding author upon request.

## Supplementary Materials

Supplementary Materials Table S1: rates of mitochondrial respiration. Figure S1: amounts of respiratory chain complex I subunits. Figure S2: amounts of respiratory chain complex III and IV subunits. Figure S3: amounts of respiratory chain complex V subunits. Figure S4: amounts of citrate cycle enzymes and their subunits. Figure S5: amounts of fatty acid beta-oxidation enzymes. Figure S6: amounts of glycolytic enzymes.
