# Supplementary material for: Increased Mitochondrial Protein Levels and Bioenergetics in the Musculus Rectus Femoris of Wfs1-Deficient Mice
Source: Oxid Med Cell Longev. 2018 Nov 21;2018:3175313. doi: 10.1155/2018/3175313 (PMC6280240; doi:10.1155/2018/3175313)
Supplement: Supplementary Materials — Table S1: rates of mitochondrial respiration. Figure S1: amounts of respiratory chain complex I subunits. Figure S2: amounts of respiratory chain complex III and IV subunits. Figure S3: amounts of respiratory chain complex V subunits. Figure S4: amounts of citrate cycle enzymes and their subunits. Figure S5: amounts of fatty acid beta-oxidation enzymes. Figure S6: amounts of glycolytic enzymes. [file 3175313.f1.pdf]

Supplementary Information

Increased Mitochondrial Protein Levels and Bioenergetics in the Musculus Rectus Femoris of Wfs1-Deficient Mice

Margus Eimre[a] , Kalju Paju[a], Nadežda Peet[a], Lumme Kadaja[a], Marian Tarrend[a], Sergo Kasvandik[b], Joosep Seppet[c], Marilin Ivask[a], Ehte Orlova[a], Sulev Kõks[a]

[a] Chair of Pathological Physiology, Institute of Bio- and Translational Medicine, University of Tartu, Ravila 19, 50411 Tartu, Estonia

[b] Proteomics Core Facility, Institute of Technology, University of Tartu, Nooruse 1, 50411 Tartu, Estonia

[c] Department of Pathology, Tartu University Hospital, L. Puusepa 8, 51014 Tartu, Estonia

Parameter of mitochondrial respiration	*m. rectus femoris*		*m. soleus*		heart	
	+/+	-/-	+/+	-/-	+/+	-/-
V_0	0.12±0.02<br>n = 9	0.31±0.03 ***<br>n = 5	0.36±0.07<br>n = 7	0.28±0.04<br>n = 7	1.12±0.09<br>n = 8	1.02±0.07<br>n = 8
V_{ADP}	1.06±0.10<br>n = 9	1.48±0.13 *<br>n = 8	2.51±0.21<br>n = 7	1.66±0.11 *<br>n = 7	6.25±0.70<br>n = 8	5.02±0.36<br>n = 8
V_{Rot}	0.07±0.01<br>n = 9	0.11±0.02<br>n = 8	0.06±0.09<br>n = 7	0.06±0.03<br>n = 7	0.52±0.08<br>n = 8	0.48±0.05<br>n = 8
V_{Succ}	0.70±0.09<br>n = 9	1.13±0.09 **<br>n = 8	1.91±0.13<br>n = 7	1.42±0.11 *<br>n = 7	7.46±0.76<br>n = 8	6.38±0.45<br>n = 8
V_{Atr}	0.31±0.04<br>n = 9	0.54±0.04 ***<br>n = 8	0.88±0.07<br>n = 7	0.65±0.06<br>n = 7	2.75±0.19<br>n = 8	2.46±0.11<br>n = 8
V_{Ant}	0.09±0.02<br>n = 9	0.15±0.02<br>n = 8	0.31±0.10<br>n = 7	0.16±0.04<br>n = 7	0.84±0.10<br>n = 8	0.74±0.08<br>n = 8
V_{TMPD}	1.90±0.14<br>n = 9	2.35±0.22 *<br>n = 8	3.85±0.29<br>n = 7	3.73±0.47<br>n = 7	16.44±2.22<br>n = 8	14.60±1.60<br>n = 8
V_{NaN3}	0.65±0.06<br>n = 9	0.83±0.10<br>n = 8	1.58±0.15<br>n = 7	1.76±0.24<br>n = 7	2.99±0.17<br>n = 8	2.76±0.12<br>n = 8
V_{ADP} -V_0	0.94±0.08<br>n = 9	1.17±0.12<br>n = 8	2.15±0.22<br>n = 7	1.38±0.13 *<br>n = 7	5.13±0.69<br>n = 8	4.01±0.32<br>n = 8
V_{ADP}/V_0	10.60±1.18<br>n = 9	4.87±0.44 ***<br>n = 8	9.30±2.90<br>n = 7	8.63±3.39<br>n = 7	5.84±0.75<br>n = 8	5.06±0.46<br>n = 8

Table S1. Rates of mitochondrial respiration. Values are means ± SE; n - the number of specimens studied. The rates of respiration (V) are given in nmol O_2/min/mg wet weight. V_0, basal respiration without ADP; V_{ADP}, ADP-stimulated respiration; $V_{Rot,}$ respiration in the presence of rotenone; V_{Succ}, ADP-stimulated respiration in the presence of rotenone and succinate; V_{Atr}, respiration after inhibition of succinate and ADP-stimulated respiration by atractyloside; V_{Ant}, respiration in the presence of AntA; V_{TMPD}, TMPD+ascorbate stimulated respiration; V_{NaN3}, TMPD-stimulated respiration after addition of NaN3; V_{ADP}-V_0, OXPHOS dependent respiration; V_{ADP}/V_0, respiration control index; * $p < 0.05$, ** $p < 0.01$, *** $p < 0.001$ compared with wild type.

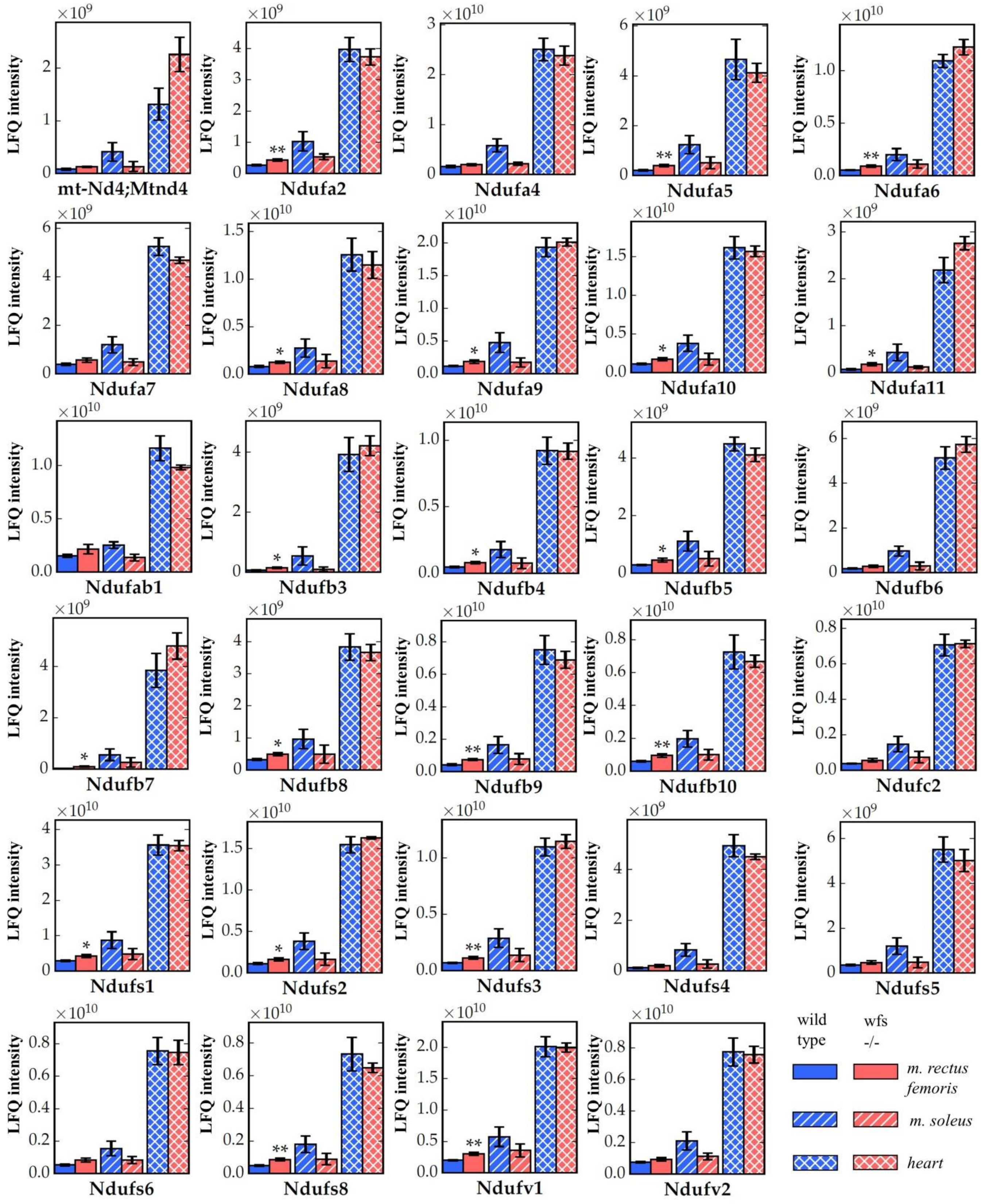

Figure S1. By LC/MS/MS analysis determined amounts of respiratory chain complex I subunits. Mtnd4 – NADH-ubiquinone oxidoreductase chain 4, Ndufa – NADH dehydrogenase [ubiquinone] 1 alpha subcomplex subunits, Ndufb - NADH dehydrogenase [ubiquinone] 1 beta subcomplex subunits, Ndufc2 - NADH dehydrogenase [ubiquinone] 1 subunit C2, Ndufs – NADH dehydrogenase [ubiquinone] iron-sulfur proteins, Ndufv - NADH dehydrogenase [ubiquinone] flavoprotein; * - $p<0.05$, ** - $p<0.01$ compared to wild type.

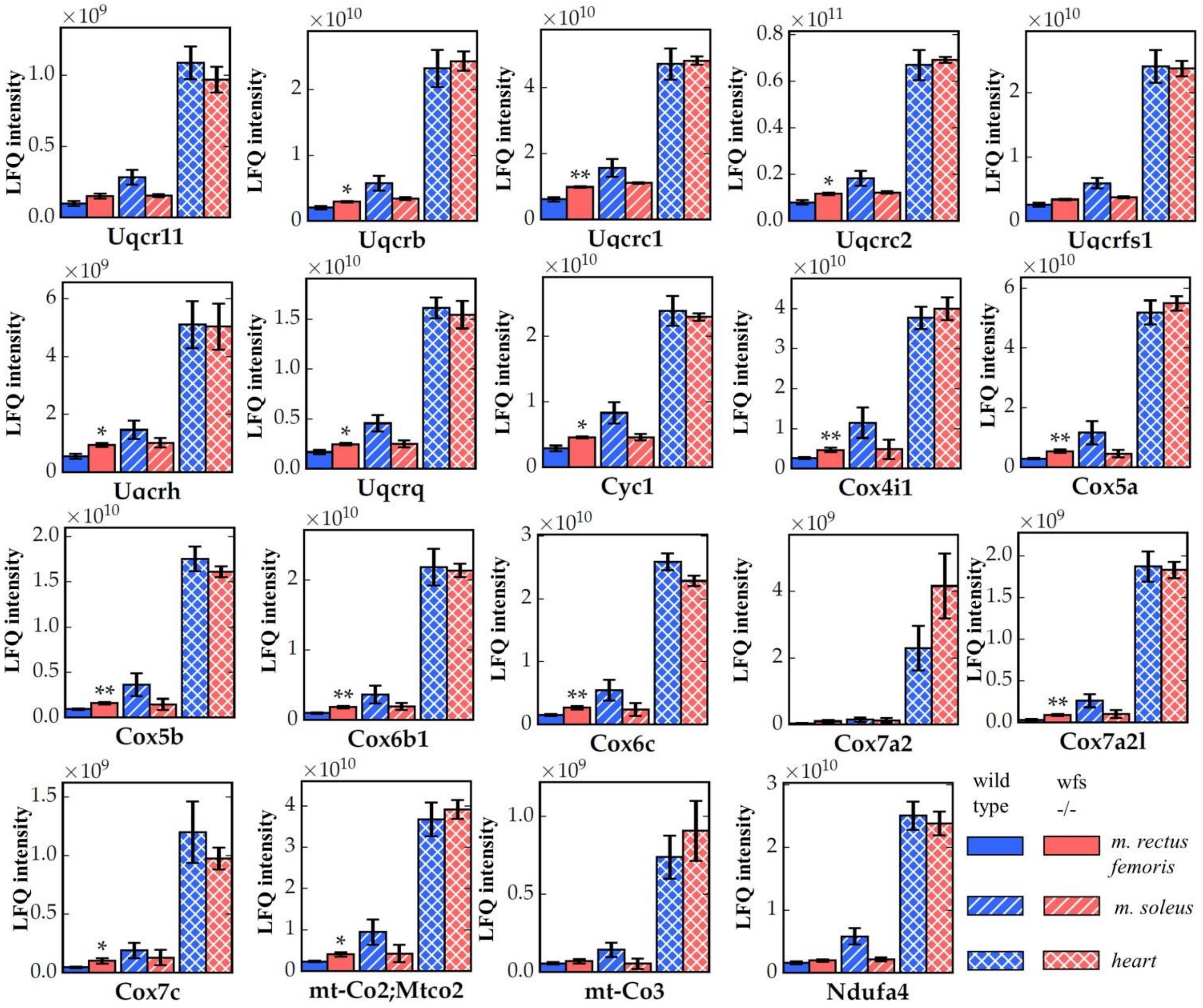

Figure S2. By LC/MS/MS analysis determined amounts of respiratory chain complexis III and IV subunits. Uqcr11 – Cytochrome b-c1 complex subunit 10, Uqcrb – Cytochrome b-c1 complex subunit 7, Uqcrc1 – Cytochrome b-c1 complex subunit 1, Uqcrc2 - Cytochrome b-c1 complex subunit 2, Uqcrfs1 – Cytochrome b-c1 complex subunit Rieske, Uqcrh – Cytochrome b-c1 complex subunit 6, Uqcrq – Cytochrome b-c1 complex subunit 8, Cyc1 – Cytochrome c1, heme proteiin, Cox4i1 – Cytochrome c oxidase subunit 4 isoform 1, Cox5a – Cytochrome c oxidase subunit 5A, Cox5b – Cytochrome c oxidase subunit 5B, Cox6b1 – Cytochrome c oxidase subunit 6B1, Cox6c – Cytochrome c oxidase subunit 6C, Cox7a2 – Cytochrome c oxidase subunit 7A2, Cox7a2l – Cytochrome c oxidase subunit 7A-related proteiin, Cox7c – Cytochrome c oxidase subunit 7C, mt-Co2 – Cytochrome c oxidase subunit 2, mt-Co3 – Cytochrome c oxidase subunit 3, Ndufa4 – Cytochrome c oxidase subunit NDUFA4; * - $p<0.05$, ** - $p<0.01$ compared to wild type.

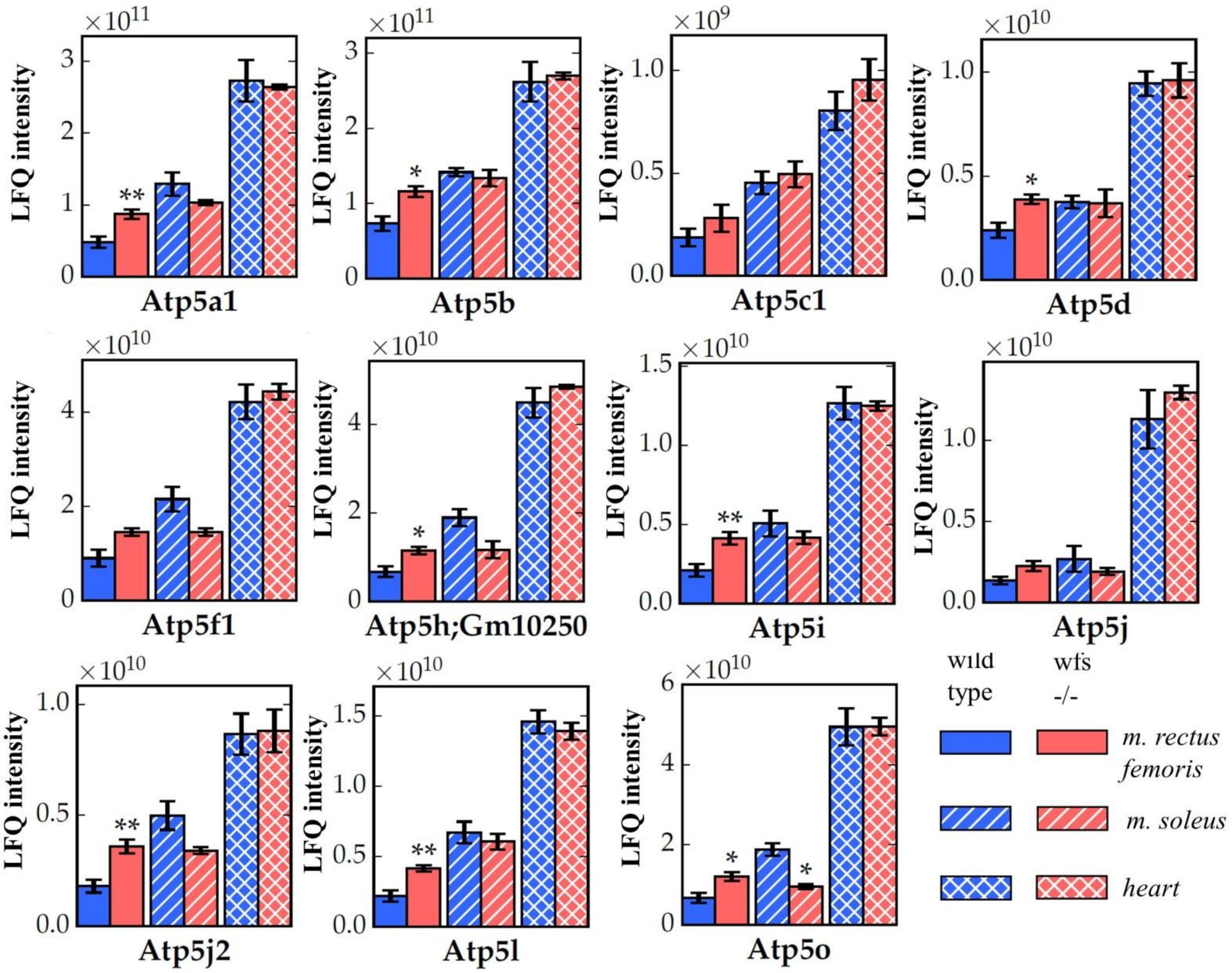

Figure S3. By LC/MS/MS analysis determined amounts of respiratory chain complex V subunits. Atp5a1 – ATP synthase subunit alpha, Atp5b – ATP synthase subunit beta, Atp5c1 – ATP synthase subunit gamma, Atp5d – ATP synthase subunit delta, Atp5f1 – ATP synthase F(0) complex subunit B1, Atp5h – ATP synthase subunit d, Atp5i – ATP synthase subunit e, Atp5j – ATP synthase-coupling factor 6, Atp5j2 – ATP synthase subunit f, Atp5l – ATP synthase subunit g, Atp5o – ATP synthase subunit O; * - $p<0.05$, ** - $p<0.01$ compared to wild type.

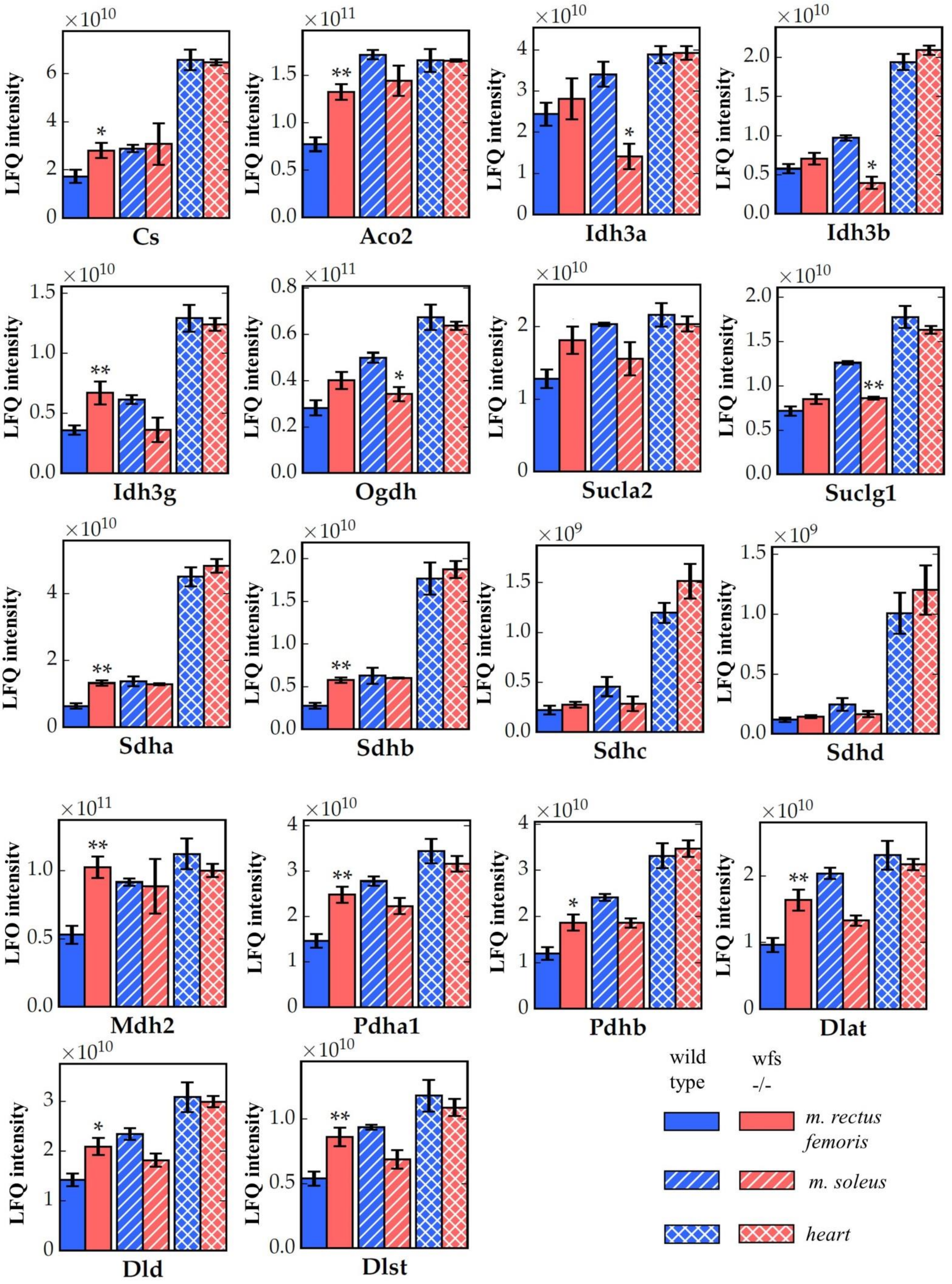

Figure S4. By LC/MS/MS analysis determined amounts of Citrate cycle enzymes and their subunits. Cs – Citrate synthase, Aco2 – Aconitate hydratase, Idh3a – Isocitrate dehydrogenase [NAD] subunit alpha, Idh3b – Isocitrate dehydrogenase [NAD] subunit, Idh3g – Isocitrate dehydrogenase [NAD] subunit gamma 1, Ogdh – 2-oxoglutarate dehydrogenase, Sucla2 – Succinyl-CoA ligase [ADP-forming] subunit beta, Suclg1 – Succinyl-CoA ligase [ADP/GDP-forming] subunit alpha, Sdha – Succinate dehydrogenase [ubiquinone] flavoprotein subunit, Sdhb – Succinate dehydrogenase [ubiquinone] iron-sulfur subunit, Sdhc- Succinate dehydrogenase cytochrome b560 subunit, Sdhd – Succinate dehydrogenase [ubiquinone] cytochrome b small subunit, Mdh2 – Malate dehydrogenase, Pdha1 – Pyruvate dehydrogenase E1 component subunit alpha, Pdhb – Pyruvate dehydrogenase E1 component subunit beta , Dlat – Dihydrolipoyllysine-residue acetyltransferase component of pyruvate dehydrogenase complex, Dld – Dihydrolipoyl dehydrogenase, Dlst – Dihydrolipoyllysine-residue succinyltransferase component of 2-oxoglutarate dehydrogenase complex; * - $p<0.05$, ** - $p<0.01$ compared to wild type.

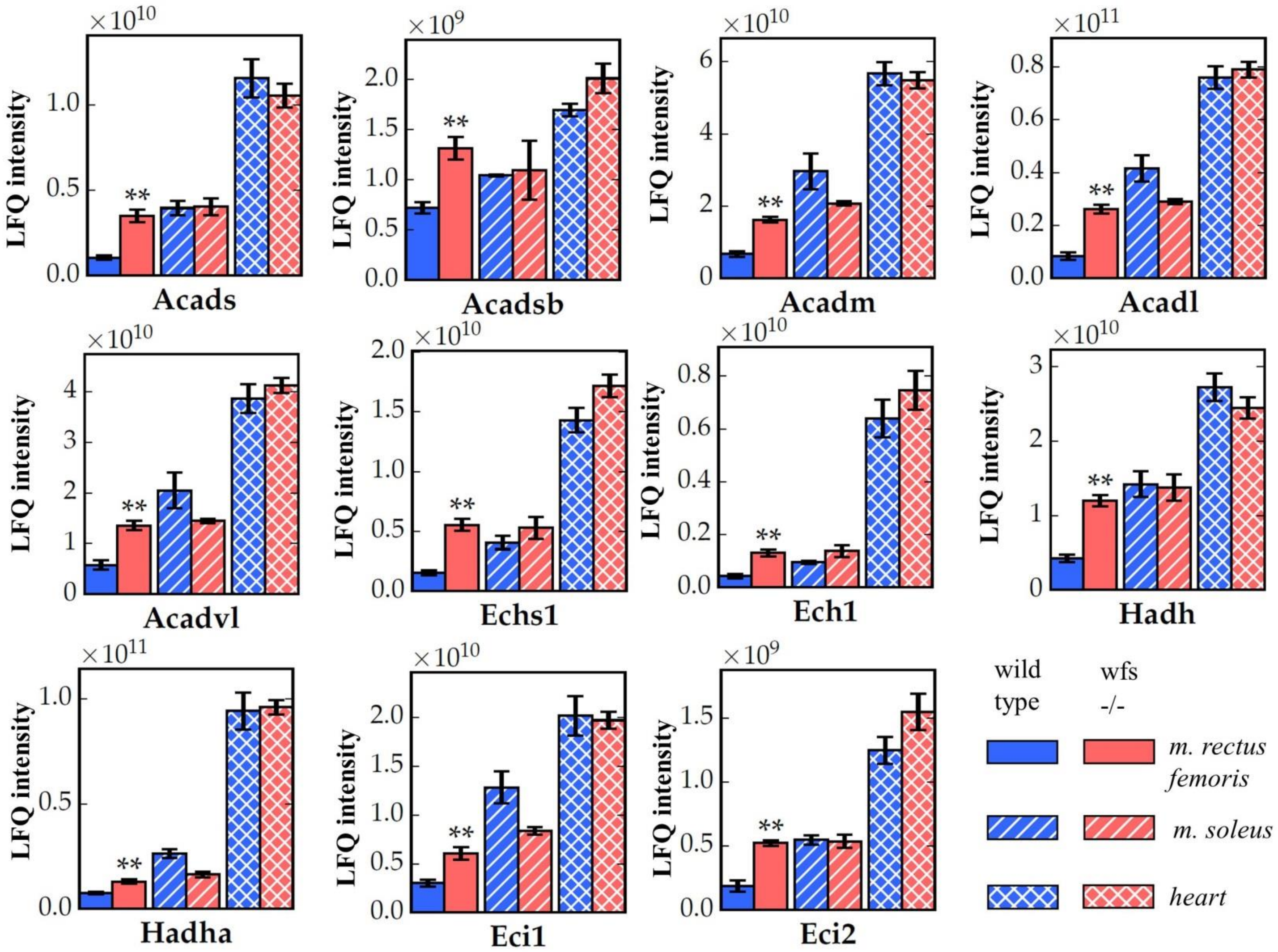

Figure S5. By LC/MS/MS analysis determined amounts of fatty acids beta-oxidation enzymes. Acads - short-chain specific acyl-CoA dehydrogenase Acadsb – short/branced chain specific acyl-CoA dehydrogenase, Acadm - medium-chain specific acyl-CoA dehydrogenase, Acadl - long-chain specific acyl-CoA dehydrogenase, Acadvl – very-long chain specific acyl-CoA dehydrogenase, Echs1 - Enoyl-CoA hydratase, Ech1 - Delta(3,5)-Delta(2,4)-dienoyl-CoA isomerase, Hadh - hydroxyacyl-coenzyme A dehydrogenase, Hadha - trifunctional enzyme subunit alpha, Eci1 - Enoyl-CoA delta isomerase 1, Eci2 - Enoyl-CoA delta isomerase 2 ; * - $p<0.05$, ** - $p<0.01$ compared to wild type.

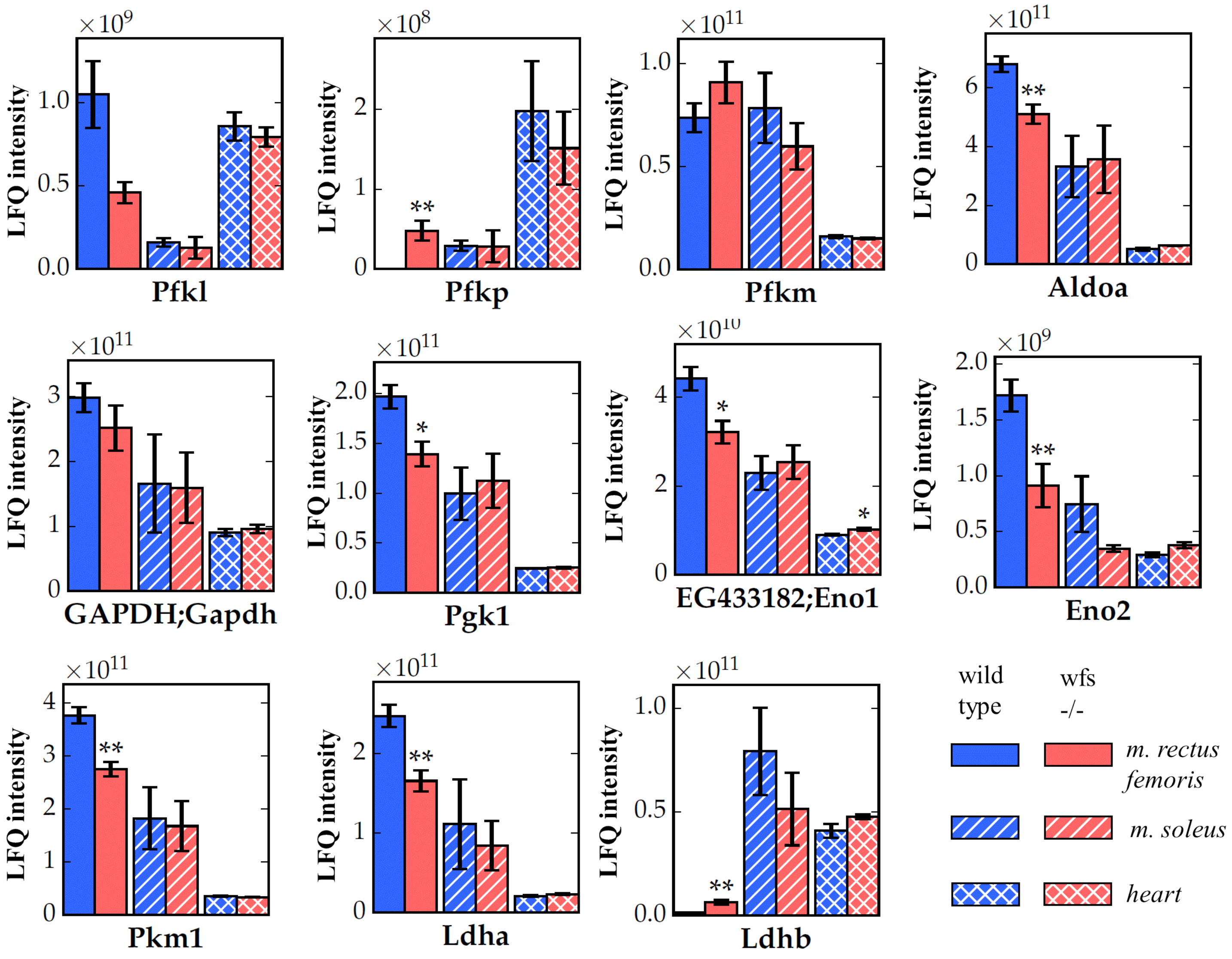

Figure S6. By LC/MS/MS analysis determined amounts of glycolytic enzymes. Pfkl – ATP-dependent 6-phosphofructokinase, liver type, Pfkp – ATP-dependent 6-phosphofructokinase, platelet type, Pfkm – ATP-dependent 6-phosphofructokinase, muscle type, Aldoa – Fructose-bisphosphate aldolase, Gapdh – Glyceraldehyde-3-phosphate dehydrogenase, Pgk1 – Phosphoglycerate kinase, G433182;Eno1 – Alpha-enolase, Eno2 – Gamma-enolase, Ldha – L-lactate dehydrogenase A chain, Ldhb –lactate dehydrogenase B chain; * - $p<0.05$, ** - $p<0.01$ compared to wild type.
